# Localization of secreted ferritin (FER2) in the embryos of the tick *Haemaphysalis longicornis*

**DOI:** 10.1186/s13071-023-05669-5

**Published:** 2023-01-30

**Authors:** Emmanuel Pacia Hernandez, Kei Shimazaki, Hiroko Niihara, Rika Umemiya-Shirafuji, Kozo Fujisaki, Tetsuya Tanaka

**Affiliations:** 1 Department of Veterinary Paraclinical Sciences, College of Veterinary Medicine, University of the Philippines at Los Baños College, 3004 Laguna, Philippines; 2 Laboratory of Infectious Diseases, Joint Faculty of Veterinary Medicine, Kagoshima University, 1-21-24 Korimoto, Kagoshima, 890-0056 Japan; 3 National Research Center for Protozoan Diseases, Obihiro University of Agriculture and Veterinary Medicine, Obihiro, Hokkaido 080-8555 Japan; 4 National Agricultural and Food Research Organization, 3-1-5 Kannondai, Tsukuba, Ibaraki 305-0856 Japan

## Abstract

Despite the absence of a blood meal, embryogenesis involves many processes that require nutrients and other essential elements, including iron. Due to the lack of an external source of these nutrients, these requirements are acquired maternally. Because of the toxic nature of iron, they are transferred through iron transport molecules such as secreted ferritin (FER2). Here we tried to follow the trail of the FER2 through indirect immunofluorescence, and we observed an apparent shift of FER2 from the germ layer at the early part of development to the appendages during the late stage of embryogenesis. FER2 is also found in the middle part of the legs of the embryo. The apparent movement not only sheds light on iron processing events during embryogenesis but also indirectly guides organogenesis in the tick.

**Graphical Abstract:**

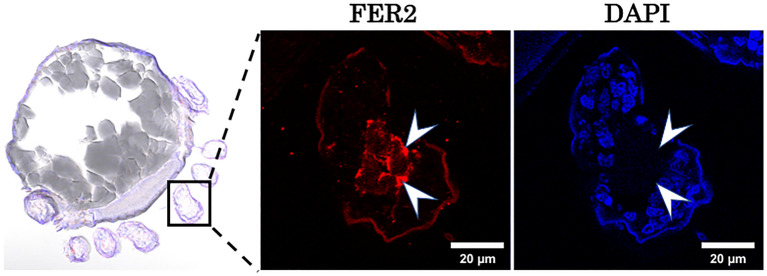

**Supplementary Information:**

The online version contains supplementary material available at 10.1186/s13071-023-05669-5.

## Background

*Haemaphysalis longicornis* is an ixodid tick belonging to the genus *Haemaphysalis*. In addition, it is a blood-sucking arthropod that acts as a vector of various disease-causing pathogens such as *Theileria orientalis* and *Babesia ovata* [1–3]. It can also serve as a reservoir of the severe fever with thrombocytopenia syndrome (SFTS) virus that can affect humans [4]. These signify the tick’s veterinary and public health importance. In Japan, it is distributed from Hokkaido to Okinawa Prefecture; overseas, it is widely distributed from Asia to Australia and New Zealand. In recent years, reports of *H. longicornis* in North America have been confirmed [3, 5].

Ticks suck blood at all developmental stages except for the egg stage to acquire nutrients for metamorphosis and reproduction [6, 7]. In embryogenesis, energy is obtained from the yolk protein vitellin, lipids, and glycogen [8, 9]. The utilization of oxygen molecules in such energy production can generate reactive oxygen species (ROS) and cause cell damage. Therefore, an antioxidant system is needed to protect embryos from ROS [10].

Iron ions are required in many metabolic processes, such as oxygen transport, oxidative phosphorylation in mitochondria, and DNA replication; they are essential trace elements for living organisms [11]. On the other hand, excess ferrous ions have been shown to catalyze the Fenton reaction that generates hydroxyl radicals from hydrogen peroxide, damaging cells [12]. Ferritin (FER) is an iron-binding protein involved in iron metabolism in almost all organisms [13]. There are two types of *H. longicornis* FER—intracellular FER1 and secreted FER2 [12, 14]. Both convert excess divalent iron ions into nontoxic trivalent iron ions and have an antioxidant effect. In particular, FER2 has also been reported to function as an iron transport protein that transports iron ions from the midgut to the ovaries [14–16]. Interestingly, knockdown of the *FER* genes has been reported to reduce tick production and hatchability [12]. Therefore, it is suggested that FERs may play an important role in embryogenesis. However, it remains unclear whether FER2 transported from the midgut to the ovary is retained in developing embryos. Thus, this experiment aims to establish the localization of FER2 during embryogenesis.

## Methods

### Ticks and experimental animals

Four-week-old female Japanese white rabbits (KBT Oriental, Japan) were used to feed blood and subculture the parthenogenetic *H. longicornis* (Okayama strain). The ticks and rabbits used in the experiments were kept in accordance with the maintenance protocol set by the Laboratory of Infectious Diseases, Joint Faculty of Veterinary Medicine, Kagoshima University, in accordance with the guidelines set by the Animal Care and Use Committee of Kagoshima University (approval number VM 18056).

### Deshelling and fixing tick eggs

Tick eggs were collected and treated in accordance with the protocols established by Santos et al. with some modifications [17]. Briefly, the tick eggs were transferred to a 1.5-ml tube according to the number of days (1st, 5th, 10th, 15th, and 20th days) elapsed after laying. We then washed the eggs with 1.5% sodium hypochlorite and 5% sodium carbonate, followed by washing with sterile water three times for 5 min each. To remove the eggshell, 1 ml of water was added and placed in a heat block at 90 °C for 2 min, and then immediately transferred to ice and allowed to stand for 2 min to crack the egg shells. Next, a fixation solution containing 4% paraformaldehyde in phosphate-buffered saline (PBS) and heptane was added at a ratio of 1:1 and mixed by inversion for 1 h at room temperature. After that, the lower layer containing the paraformaldehyde was removed, 100% methanol was added, and the tube was vigorously shaken. Eggs without their shells sank toward the bottom of the tube. The embryos were then fixed by washing with 100% methanol three times for 5 min each, followed by washing with PBS three times for 5 min each. The eggs were then inverted and mixed at 4 °C for 24–48 h in a 20% sucrose solution.

### Localization analysis of FER2 protein by indirect immunofluorescence

Embryos collected from the above procedure were embedded in OCT compound (Sakura Fintech Japan, Japan) and then allowed to stand at −80 °C to make a frozen block. Embryo sections were prepared using a Leica CM1850 cryostat (Leica Biosystems, Germany). The prepared embryo sections were attached to a cryofilm (Section-Lab, Japan) and air-dried, and then the surface of the cryofilm was covered with a 3% skim milk solution (blocking solution) prepared in PBS and incubated at room temperature for 1 h. Next, the surface of the cryofilm was covered with an anti-FER2 [12] antibody diluted 100-fold with a blocking solution and incubated at 4 °C overnight. The next day, the surface of the cryofilm was covered with goat Alexa Fluor 594-labeled anti-mouse immunoglobulin G (IgG) antibodies (Life Technologies, USA) diluted 100-fold with a blocking solution and incubated at room temperature for 1 h in the dark. Next, VECTASHIELD mounting medium with DAPI (Vector Laboratories, USA) was added dropwise and reacted at 4 °C for 1 h for nuclear staining. Several sections at the different embryonic development stages were observed using an LSM 700 confocal laser scanning microscope (Carl Zeiss, Switzerland).

## Results and discussion

Embryogenesis refers to the process leading to the formation of tissues and organs by precisely controlling cell proliferation, differentiation, and apoptosis [10]. The yolk protein vitellin is used as an energy source in tick embryogenesis, while adenosine triphosphate (ATP) is synthesized via the electron transport chain and oxidative phosphorylation in mitochondria [8]. However, the use of oxygen in the process of ATP synthesis can generate reactive oxygen species (ROS), causing damage to biopolymers such as nucleic acids, proteins, and lipids and inducing abnormal embryogenesis. Therefore, a defense mechanism is needed to protect germ cells from ROS [10]. ROS can also be generated from the Fenton reaction from ferrous iron. In other tick stages, these iron ions are acquired from a blood meal. However, in embryos, iron ions are transferred from female ticks to eggs and are used as an important nutrient source for embryogenesis. Because of this, the iron ions in embryos are limited. Therefore, iron-transporting proteins are required to transport the required amount to each organ [18]. Such iron transport proteins include transferrin and FER2, but transferrin is not detected in *H. longicornis* embryos [19]. Therefore, it is possible that the FER2 protein functions as a major iron transport protein in embryos

Our previous study analyzed the expression dynamics of FER2 in *H. longicornis* to understand the antioxidant system during embryogenesis [7]. Gene expression analysis revealed increasing *FER2* gene expression, especially on day 1 and day 5 post-oviposition, which gradually increased on day 10 and reached the highest expression on day 15. On the other hand, the protein expression remained constant throughout. Supporting this, the ferrous iron proportion was maintained throughout embryogenesis. This indicates the need for the careful maintenance of FER2 throughout embryogenesis and its crucial role in maintaining iron homeostasis during embryogenesis.

**Fig. 1:**
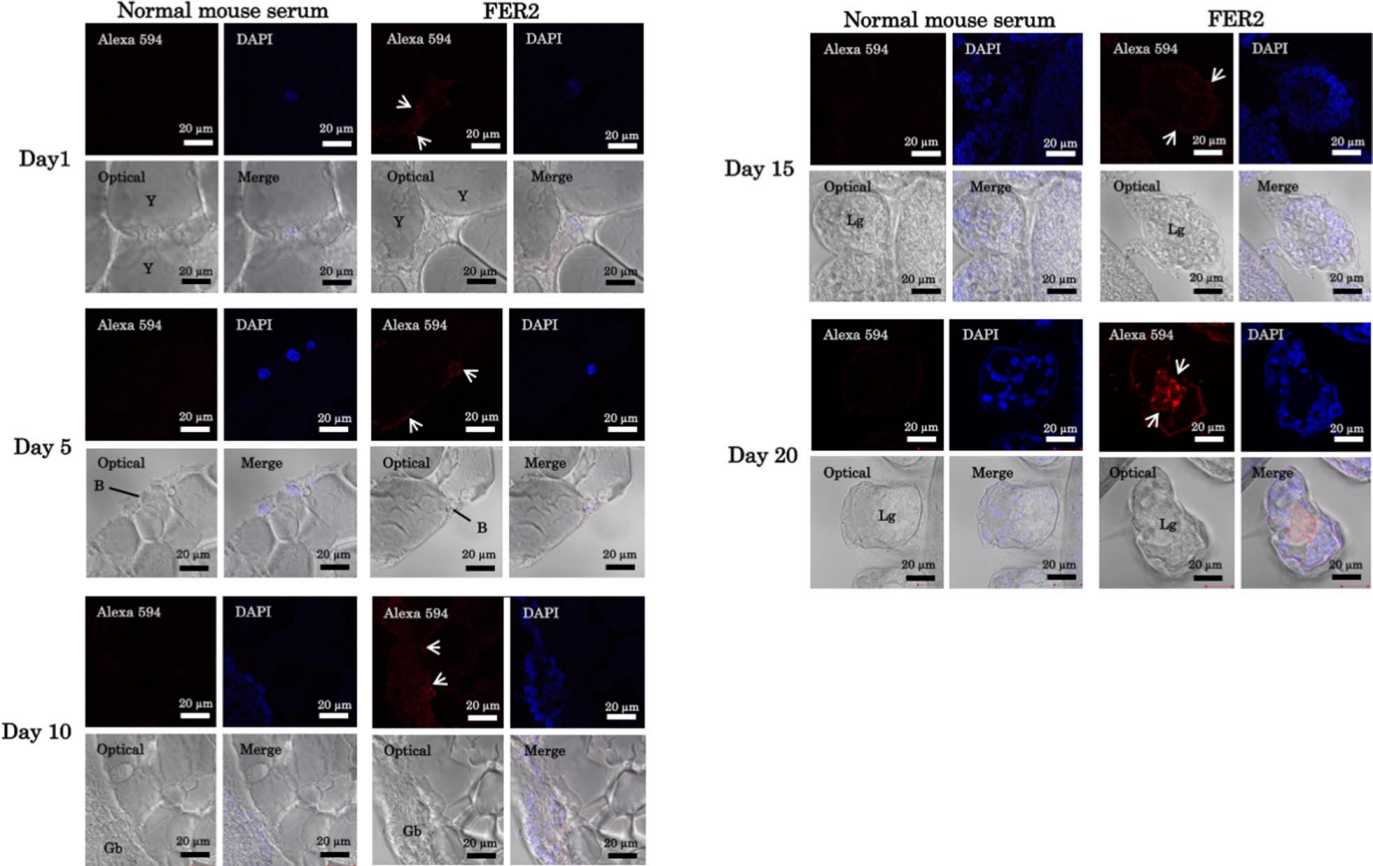
Localization of FER2 in *Haemaphysalis longicornis* during embryogenesis using the indirect immunofluorescence antibody test (IFAT). Normal mouse serum was used as a control. Arrows indicate FER2 protein fluorescence. *Y* yolk protein, *B* scutellum, *Gb* germ band, *Lg* leg

**Fig. 2:**
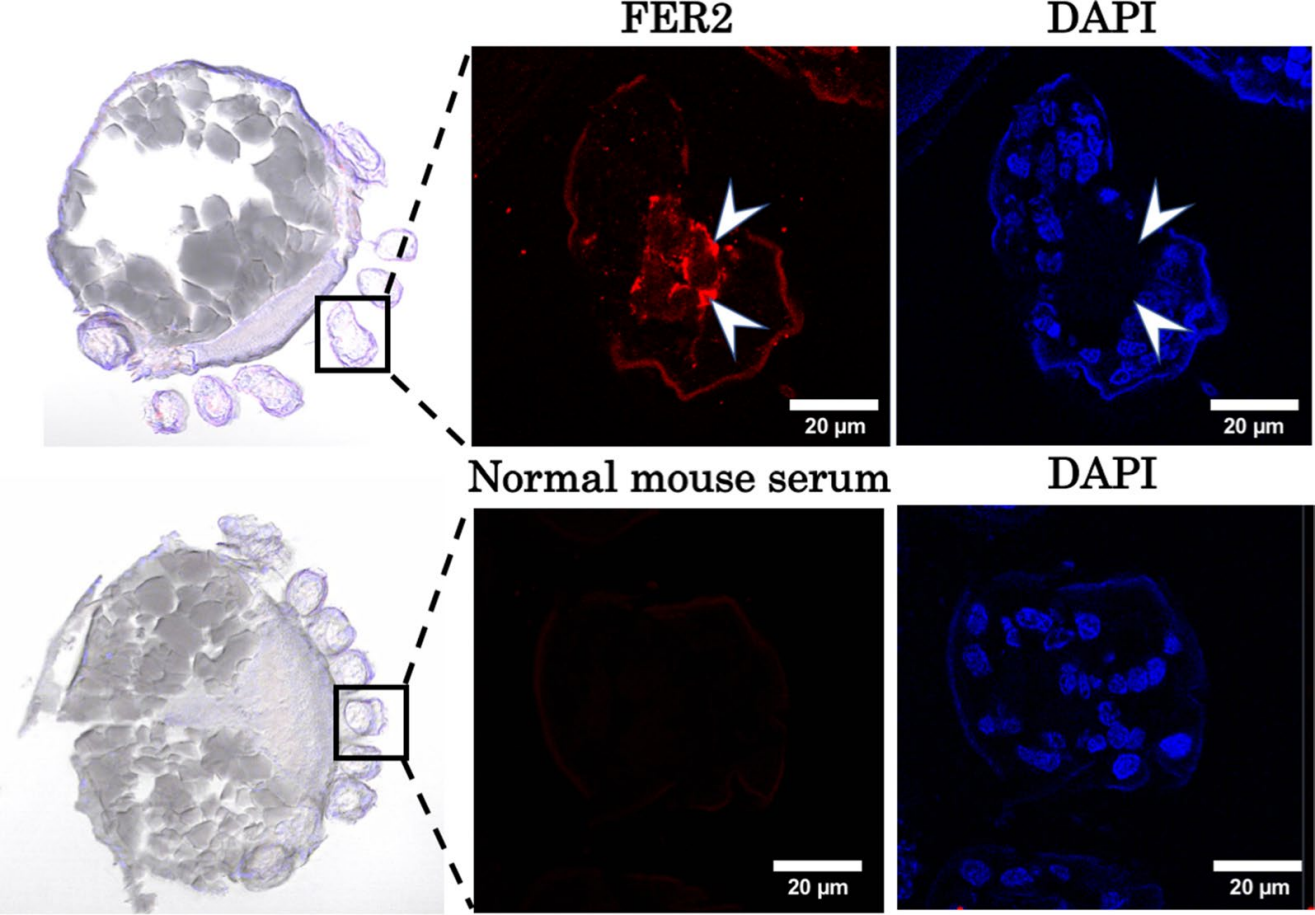
Localization of FER2 in *Haemaphysalis longicornis* embryo sections at day 20 post-oviposition. Normal mouse serum was used as a control. Arrows indicate the FER2 protein localization as determined by the immunofluorescence antibody test (IFAT)

To further understand this role, we identified the localization of FER2 proteins during embryogenesis using indirect immunofluorescence (Fig. 1) (Additional file 1). FER2 fluorescence was observed in the cytoplasm of germ cells during the entire duration of embryogenesis from the first day to the 20th. However, on the 20th day, strong fluorescence was also observed in the central part of the leg (Fig. 2). These findings indicate that FER2 is secreted into the central part of the leg on the 15th and 20th days, contributing to iron transport and maintenance of iron ion concentration in those organs. This coincides with the findings that organogenesis occurs during day 15 post-oviposition of *Hyalomma dromedarii* [20]. When organogenesis occurs, endogenous FER2 can already be produced by the gut cells. In adult ticks, FER2 produced by the gut cells is distributed to other organs with the hemolymph during blood-feeding [12]. However, during embryogenesis, it can be assumed that the cavities for the hemolymph are formed late; therefore, during the early stages of embryogenesis, FER2 is dispersed throughout the embryo. The concentration of FER2 protein in the center of the legs signifies the hollowing of those leg portions, concentrating the FER2 proteins in those areas, and may indicate the presence of hemolymph. Unlike in its adult counterpart, wherein FER2 protein is conserved only in the hemolymph during blood-feeding [12], in the embryo, FER2 may be circulating already during embryogenesis as an iron transporter to the different cells of the tick. Further experiments may be necessary to prove this claim; however, the findings herein indicate a new perspective in hemolymph development and the hollowing of other organs such as the legs. Furthermore, this could also indicate that FERs are important not only in preventing iron toxicity, like in their adult counterpart. But during embryogenesis, FER is utilized not only for the safe distribution of iron but also for its conservation, as no significant increase in the toxic ferrous iron concentration was observed, nor was the stimulation of the FER1 protein production controlled by the ferrous iron concentration [7, 21]. This could indicate the need for the iron to be safely conserved not only during embryogenesis but also during the metabolic processes of the larval stage of the tick, as the ticks assimilate no new fresh iron. The findings further deepen the knowledge of iron metabolism and oxidative stress management during embryogenesis. And for the tick and tick-borne disease control perspective, the results herewith support the targeting of oxidative stress-related molecules such as FER2 for tick vaccine or gene manipulation strategy.

## Supplementary Information

**Additional file 1. **Localization of FER2 in *Haemaphysalis longicornis* during embryogenesis using the indirect immunofluorescence antibody test (IFAT) at ×20 magnification. Normal mouse serum was used as a control. Arrows indicate FER2 protein fluorescence. *Y* yolk protein, *B* scutellum, *Gb* germ band, *Lg* leg.

## Data Availability

All data generated or analyzed during this study are included in this published article and its supplementary information file.

## References

[1] Sivakumar T, Igarashi I, Yokoyama N (2016). *Babesia ovata*: taxonomy, phylogeny and epidemiology. Vet Parasitol.

[2] Tsuji N, Miyoshi T, Battsetseg B, Matsuo T, Xuan X, Fujisaki K (2008). A cysteine protease is critical for *Babesia* spp. transmission in Haemaphysalis ticks. PLoS Pathog.

[3] Sonenshine DE, Roe RM (2014). Biology of ticks.

[4] Luo L-M, Zhao L, Wen H-L, Zhang Z-T, Liu J-W, Fang L-Z, Xue Z-F, Ma D-Q, Zhang X-S, Ding S-J, Lei X-Y, Yu X (2015). *Haemaphysalis longicornis* ticks as reservoir and vector of severe fever with thrombocytopenia syndrome virus in China. Emerging Infect Dis.

[5] Raghavan RK, Barker SC, Cobos ME, Barker D, Teo EJM, Foley DH, Nakao R, Lawrence K, Heath ACG, Peterson AT (2019). Potential spatial distribution of the newly introduced long-horned tick, *Haemaphysalis longicornis* in North America. Sci Rep.

[6] Kusakisako K, Masatani T, Miyata T, Galay RL, Maeda H, Talactac MR, Tsuji N, Mochizuki M, Fujisaki K, Tanaka T (2016). Functional analysis of recombinant 2-Cys peroxiredoxin from the hard tick *Haemaphysalis longicornis*. Insect Mol Biol.

[7] Hernandez EP, Shimazaki K, Niihara H, Umemiya-Shirafuji R, Fujisaki K, Tanaka T (2020). Expression analysis of glutathione S-transferases and ferritins during the embryogenesis of the tick *Haemaphysalis longicornis*. Heliyon.

[8] Raikhel AS, Dhadialla TS (1992). Accumulation of yolk proteins in insect oocytes. Annu Rev Entomol.

[9] Martins R, Ruiz N, da Fonseca RN, da Vaz Junior IS, Logullo C (2018). The dynamics of energy metabolism in the tick embryo. Rev Bras Parasitol Vet.

[10] Ufer C, Wang CC (2011). The roles of glutathione peroxidases during embryo development. Front Mol Neurosci.

[11] Hentze MW, Muckenthaler MU, Andrews NC (2004). Balancing acts: molecular control of mammalian iron metabolism. Cell.

[12] Galay RL, Aung KM, Umemiya-Shirafuji R, Maeda H, Matsuo T, Kawaguchi H, Miyoshi N, Suzuki H, Xuan X, Mochizuki M, Fujisaki K, Tanaka T (2013). Multiple ferritins are vital to successful blood feeding and reproduction of the hard tick *Haemaphysalis longicornis*. J Exp Biol.

[13] Andrews SC, Arosio P, Bottke W, Briat JF, von Darl M, Harrison PM, Laulhère JP, Levi S, Lobreaux S, Yewdall SJ (1992). Structure, function, and evolution of ferritins. J Inorg Biochem.

[14] Hajdusek O, Sojka D, Kopacek P, Buresova V, Franta Z, Sauman I, Winzerling J, Grubhoffer L (2009). Knockdown of proteins involved in iron metabolism limits tick reproduction and development. Proc Natl Acad Sci USA.

[15] Galay RL, Umemiya-Shirafuji R, Mochizuki M, Fujisaki K, Tanaka T (2015). Iron metabolism in hard ticks (Acari: Ixodidae): the antidote to their toxic diet. Parasitol Int.

[16] Galay RL, Matsuo T, Hernandez EP, Talactac MR, Kusakisako K, Umemiya-Shirafuji R, Mochizuki M, Fujisaki K, Tanaka T (2018). Immunofluorescent detection in the ovary of host antibodies against a secretory ferritin injected into female *Haemaphysalis longicornis* ticks. Parasitol Int.

[17] Santos VT, Ribeiro L, Fraga A, de Barros CM, Campos E, Moraes J, Fontenele MR, Araújo HM, Feitosa NM, Logullo C, da Fonseca RN (2013). The embryogenesis of the tick *Rhipicephalus* (*Boophilus*) *microplus*: the establishment of a new chelicerate model system. Genesis.

[18] González-Morales N, Mendoza-Ortíz MÁ, Blowes LM, Missirlis F, Riesgo-Escovar JR (2015). Ferritin is required in multiple tissues during *Drosophila melanogaster* development. PLoS ONE.

[19] Mori H, Galay RL, Maeda H, Matsuo T, Umemiya-Shirafuji R, Mochizuki M, Fujisaki K, Tanaka T (2014). Host-derived transferrin is maintained and transferred from midgut to ovary in *Haemaphysalis longicornis* ticks. Ticks Tick Borne Dis.

[20] Ibrahim MA (1998). Traffic of the tick embryo basic protein during embryogenesis of the camel tick *Hyalomma dromedarii* (Acari: Ixodidae). Exp Appl Acarol.

[21] Hernandez EP, Kusakisako K, Talactac MR, Galay RL, Yoshii K, Tanaka T (2018). Induction of intracellular ferritin expression in embryo-derived *Ixodes scapularis* cell line (ISE6). Sci Rep.

