# Supplementary figures and images for: Localization of secreted ferritin (FER2) in the embryos of the tick Haemaphysalis longicornis
Source: Parasit Vectors. 2023 Jan 30;16:42. doi: 10.1186/s13071-023-05669-5 (PMC9885654; doi:10.1186/s13071-023-05669-5)

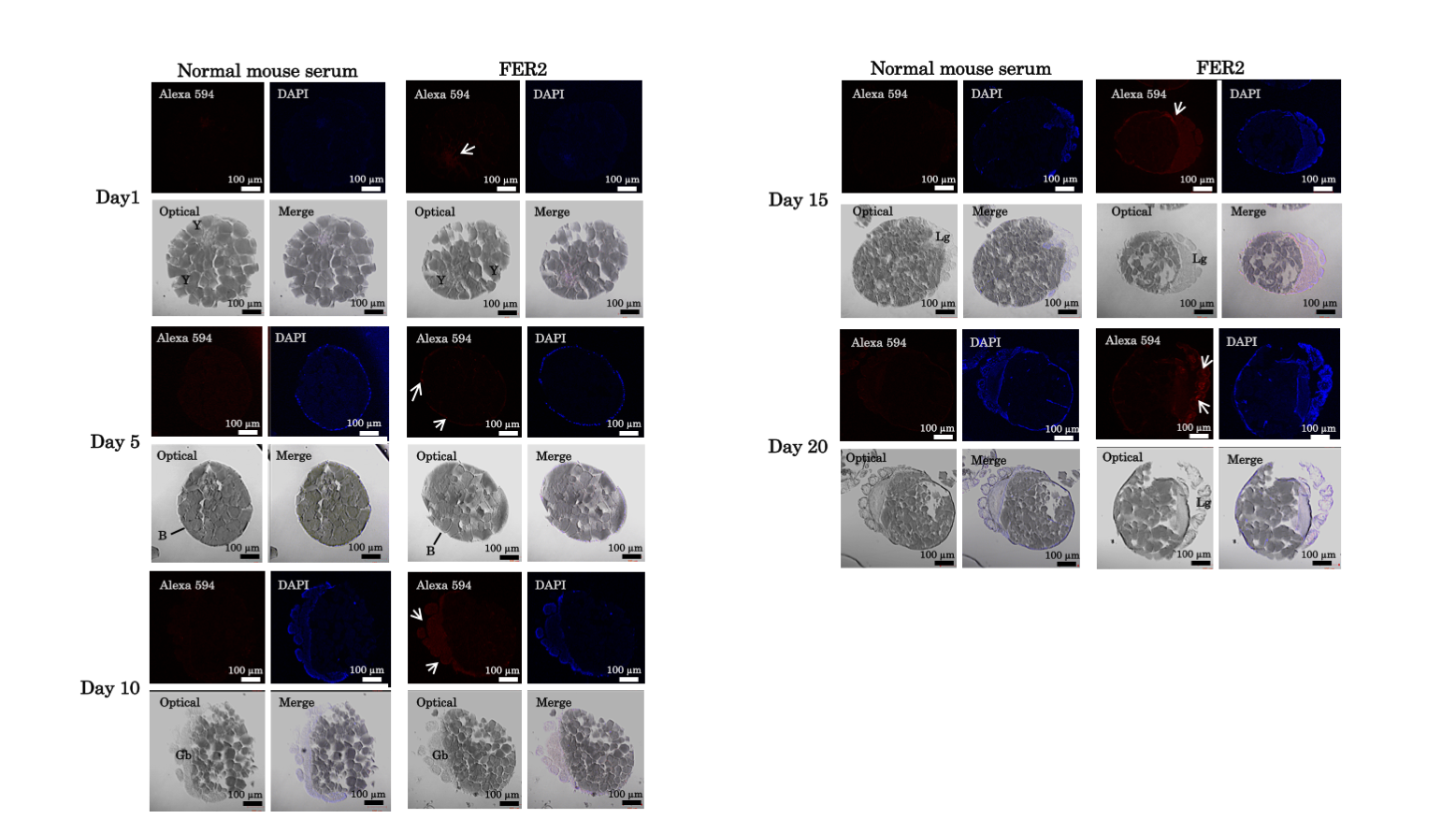

Supplement: Supplementary file 1 — Additional file 1. Localization of FER2 in Haemaphysalis longicornis during embryogenesis using the indirect immunofluorescence antibody test (IFAT) at ×20 magnification. Normal mouse serum was used as a control. Arrows indicate FER2 protein fluorescence. Y yolk protein, B scutellum, Gb germ band, Lg leg. [file 13071_2023_5669_MOESM1_ESM.tiff]
